# Consequences of *AphanizomenonFlos-aqua*e(*AFA*) extract (*Stemtech*^*TM*^) on metabolic profile of patients with type 2 diabetes

**DOI:** 10.1186/s40200-015-0177-7

**Published:** 2015-06-24

**Authors:** Maryam Sanaei, Mehdi Ebrahimi, Zahra Banazadeh, Gita Shafiee, Fatemeh Khatami, Zeinab Ahadi, Ramin Heshmat

**Affiliations:** Chronic Diseases Research Center, Endocrinology and Metabolism Population Sciences Institute, Tehran University of Medical Sciences, Tehran, Iran; Osteoporosis Research Center, Endocrinology and Metabolism Clinical Sciences Institute, Tehran University of Medical Sciences, Tehran, Iran; Endocrinology and Metabolism Research Center, Endocrinology and Metabolism Clinical Sciences Institute, Tehran University of Medical Sciences, Tehran, Iran; Lolagar hospital, Iran University of Medical Sciences, Tehran, Iran

**Keywords:** Type 2 Diabetes, CD34, *Aphanizomenonflos-aquae(AFA)*, *Stemtech*^*TM*^

## Abstract

**Background:**

Blue- green algae is one of the most nutrient dense foods which is rich in substances that have useful effects on human health. The purpose of this study was to evaluate the effectiveness of a water- soluble extract of the cyanophyta Aphanizomenon Flos-aquae (*Stemtech*^*TM*^) as a functional supplement on CD markers, lipid profile, glucose levels as well as its side effects in Iranian patients with type 2 diabetes.

**Methods:**

During this randomized, double-blind, placebo-controlled trial 49 type 2 diabetic patients, aged between 20 and 60 years with a HbA1C ≥ 7.5 %, were allocated. Patients were divided into two groups of placebo and treated with an equal ratio 1:1. The subjects in *StemtechTM* group received one capsule of *StemFlo* (508 mg) before breakfast and two capsules of *StemEnhance* (500 mg) after each meal for a period of 12 weeks, and placebo group was instructed to take placebo with the same pattern. During the intervention period, subjects were asked to keep usual diet and prohibited to take any functional foods or dietary supplements. Metabolic panel has been measured as the primary outcome of study at the beginning and end of the intervention period via blood sampling.

**Results:**

*Stemtech*^*TM*^ supplementation for 12 weeks decreased fasting blood glucose (FBG) and Glycatedhemoglobin (HbA1c). Mean serum chemistry parameters (Triglyceride, Total Cholesterol, LDL, HDL, CRP, AST, ALT, BUN and Creatinine) as well as CD 34^+^, IL-6, TNF-α in treated and control groups before and after the study showed no considerable dissimilarities.

**Conclusion:**

*StemtechTM* intervention brought in positive consequence on blood glucose levels in Iranian patients with type 2 diabetes, consequently suggests the *Stemtech*^*TM*^ as a functional food for the management of diabetes.

## Introduction

Diabetes mellitus, as a multi-factorial disease, is characterized by chronic hyperglycemia due to insulin resistance and defect in insulin secretion and/or insulin action caused by Langerhans islets β-cell failure. Other primary defects responsible for the development of diabetes are increasing in hepatic glucose production and decrease in peripheral glucose utilization [1]. It is one of the most important worldwide health problems that is estimated to affect at least 5 % of the global population by the year 2025 [2, 3]. Currently, the available therapy for diabetes includes insulin injection and various oral anti-diabetic agents such as sulfonylureas, Thiazolidinediones, α-Glucosidase inhibitors, etc. These drugs are used as monotherapy or in combination to achieve better glycaemic control. Diabetes mellitus has been classified into two forms; type 1 and type 2. Type 1 diabetes is caused by autoimmune destruction of β- cells, therefore, treatment of type 1 diabetes depends on exogenous insulin. Type 2 diabetes is more prevalent than type 1 and is considered a heterogeneous disease.

Blue- green algae is one of the most nutrient dense foods which is rich in substances that have useful effects on human health. This substance has a high concentration of vitamins, minerals and enzymes with a complete spectrum of essential and non-essential amino acids that are all easily absorbed by the body. Due to these properties, a large number of researchers were interested in employment of blue-green algae as food supplementation. Several blue-green algae, including *Aphanizomenonflos-aquae (AFA)* showed protective effects in the classical AMES test [4], antibacterial and antioxidant properties, glucose and cholesterol-regulatory effects as well as host immune system modulation [4–6]. Numerous studies have been performed to estimate the results of blue-green algae supplementation on promoting health and controlling a variety of disorders in humans [7]. There are increasing scientific and clinical evidences for its role in controlling chronic diseases such as arthritis [8] and cancer [9, 10]. This substance can enhance the phagocyte activity in macrophages [11, 12]. Moreover, inhibition of allergic reactions in rodents has been reported by this agent [13, 14]. However, there is not any authentic report about *AFA* extracts and diabetes some studies reported the lipid-lowering effect of blue-green algae in healthy [15] and diabetic patients [16].

It has been shown that blue-green algae increases the stem cells trafficking or homing in animals through induction of a transient boosting in the population of stem cells in animal’s circulatory systems [17, 18]. Two of these natural products are Stem Flo and StemEnhance (STEM Tech Health Sciences, San Clemente, CA, USA) (StemtechTM), which are extracted from AFAplant [19, 20].

Given the importance of diabetes mellitus, the purpose of this study is to evaluate the safety and efficacy of *StemtechTM* (*StemEnhance and StemFlo*) on lipid profile, glycemic control, CD markers in patients with type 2 diabetes.

## Materials and methods

This study was a double-blind, parallel randomized clinical trial (RCT) on Type 2 diabetic patients who were aged between 20 and 60 years with a Glycated hemoglobin (HbA1c) ≥ 7.5 %. Patients were allocated into two groups of treatment (StemTech group) and placebo, based on permuted balanced block randomization. Forty nine StemTech patients (9 male, 40 female) were enrolled in this study. During the follow-up period four patients (16 %) in the placebo group and three patients (12 %) inStemTech group were withdrawn which was mostly because of nausea. Subsequently the number of patients in the control group dropped to 20 in the placebo and to 22 from StemTech group. The study was approved by ethic committee and registered in the Iranian Registry of Clinical Trials (IRCT) withcode***201108074920N2***. All patients signed the informed consent form. Key inclusion criteria were set as:both sexes, aged between 18–70 years, type 2 diabetic patients with HbA1c ≥ 7.5 % and <10 %. Patients who had a systemic or chronic illness, diabetic complications such as diabetic nephropathy, malignancy, history of active inflammatory illness, were pregnant, intended to become pregnant during the study period or were breast fed excluded from the study. Moreover, patients who were on corticosteroid therapy, radiotherapy, chemotherapy or any immuno- suppressive drug, consumed alcohol, used vitamin or mineral supplements in the last 2 months, or their diabetic treatments were changed during the last two months were excluded from the study. The diagnosis of diabetes was based on American Diabetes Association Criteria [21].

Blood was drawn after a minimum of 12 h fasting at first (beginning of the intervention) and last visit (end of study) and biochemical, inflammatory and antioxidant markers were measured in collecting samples. Anthropometric parameters, nutrient intakes and quality of life were assessed at baseline.

The participants were interviewed and life-style, food expenditure, body weight, height and body mass index (BMI) were measured. The systolic and diastolic blood pressures were measured before and after study using an automatic blood pressure calculator after a 10 min rest.

The subjects in *StemTech* group received one capsule of *StemFlo* before at breakfast and two capsules of *StemEnhance* after each main meal for a period of 12 weeks. It means that they received 90 *StemFlo* (508 mg of Enzyme Blend Nattozime,Serrazime,Papain,Bromelain, Protease 6.0, Protease 4.5, Indian Gooseberry fruit, Citrus Bioflavonoid,black-currant extract, Mangos teen fruit and rind, Cats claw bark and Turmeric root extract) and 180 *StemEnhance* (500 mg *Aphanizomenonflos-aquae* extract per capsule) *(Stemtech Canada Inc).* The placebo group took placebo with the same pattern of *StemFlo* and *StemEnhance*.

The observance for all participants was completed by telephoned one every two weeks. Biochemical parameters were measured using the following materials: Malondialchehyche(MDA) (ELISA Kit, *Cat. No:E1371Hu*), Interleukin 6(IL-6) (ELISA Kit, *Cat. No:E0090Hu*) and TNF-alpha (ELISA Kit, *Cat. No: E0082Hu*). CD 34 was measured as primary outcome of study at the beginning and end of intervention period in the blood sample. The averages actual numbers of CD34 + cells were calculated by multiplying the percent CD34+ cells, based on flow-cytometry on triplicate samples from each time point.

Any adverse event, including neurotoxin activity, hepatotoxicity, allergic and skin reactions were recorded. If the adverse effect were serious enough to require medical attention, it had to be reported within a few days. Then Data and Safety Monitoring Board (DSMB) were informed and it was their duty to decide on whether or not blinding should be removed and the patient should or should not be excluded from the study.

In case of fatal or severe event requiring hospital admission, reporting should be prompted at the same day to the responsible officer in Stemtech Company by fax.

### Statistical analysis

Data were presented as mean ± SD (Standard deviation). The chi - square test was performed to determine differences at baseline in frequencies of categorized variables between the groups and mean differences were analyzed by analysis of covariance (ANCOVA). The paired *t*-test was used to analyze mean differences for all measured parameters between baseline and the end of the intervention period. To demonstrate the association between baseline blood profiles and mean change of various parameters, partial correlation coefficients analysis was conducted. Data within each group were analyzed by repeated measures analysis of variance to establish significant differences in treatment. Statistical analyses were performed with SPSS version 16.0 statistical package for Windows (SPSS Inc., Chicago, Illinois). The *P* value of less than 0.05 was considered statistically significant.

## Results

**Fig. 1:**
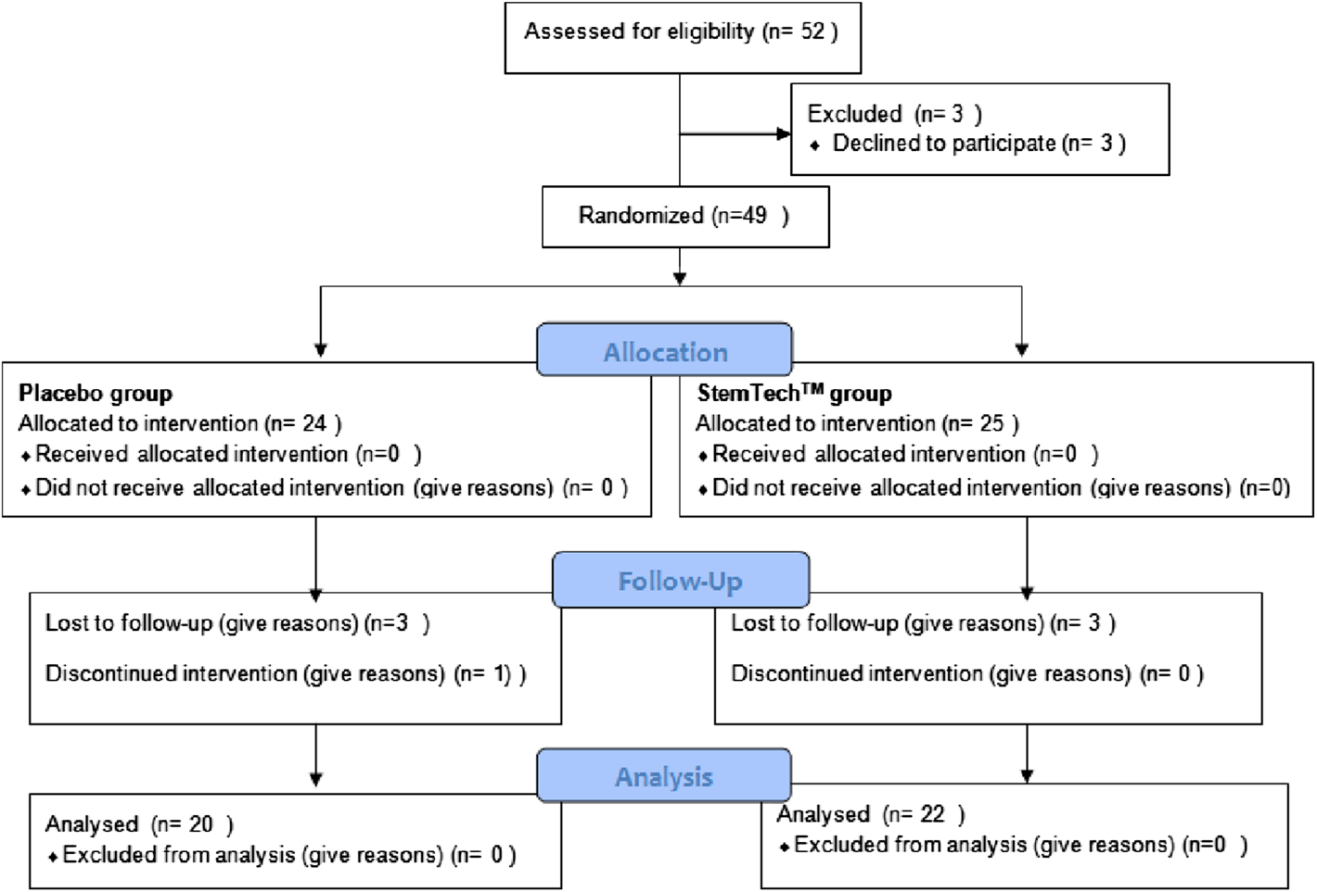
Flowchart of sample enrollment in this study

Subjects were randomly allocated to receive placebo or *StemTech*^*TM*^ for 16 weeks (Fig. 1). The duration of diabetes varied from 2 to 22 years (mean and SD:8.8 ± 5.6). The age range was from 33 to 74 years (55.4 ± 7.9 years). There was no significant difference in baseline characteristics, including age, sex, weight, BMI, waist circumference, hip circumference, duration of diabetes, systolic and diastolic blood pressure, cigarette smoking and alcohol consumption between two groups (Table 1). The biochemical parameters of the two groups also showed no significant difference in the baseline.

**Table 1:** Baseline characteristics of subjects between two studied groups

	Placebo (*n* = 24)	*StemTech* ^*TM*^ (*n* = 25)	*P*-value
Age (years)	55.7 ± 9.3	55.2 ± 6.4	0.853
Sex (Male/Female)	6/18	3/22	0.289
Weight (Kg)	77.0 ± 13.7	72.1 ± 10.8	0.178
BMI (kg/m^2^)	29.1 ± 4.8	29.0 ± 3.3	0.960
Waist circumference (cm)	104.2 ± 10.9	100.4 ± 9.8	0.347
Hip circumference (cm)	58.7 ± 12.7	55.3 ± 15.3	0.541
Duration diabetes (years)	9.0 ± 6.2	8.5 ± 5.0	0.747
Systolic blood pressure (mmHg)	12.6 ± 1.6	12.3 ± 1.6	0.653
Diastolic blood pressure (mmHg)	7.8 ± 0.7	7.4 ± 1.1	0.169
Heart rate (n)	71.7 ± 10.1	69.4 ± 10.3	0.507
Current smoking (%)	3.0 (13.0)	0.0 (0.0)	0.233
Alcohol consumption (%)	1.0 (4.3)	2.0 (8.7)	1.000

Data are mean ± Standard Deviation and number (%) where indicated

As it is shown in Table 2, no significant differences in plasma levels of MDA, TNF-a, IL-6, and CD34 percentage was observed before and after intervention in placebo and *StemTech*^*TM*^ groups.

**Table 2:** Comparison of cell marker, immunological parameters and antioxidant level before and after intervention among two studied groups

	Placebo		*P*-value	*StemTech* ^*TM*^		*P*-value
	Before	After		Before	After	
TNF-α (pg/ml)^a^	118.4 (104.8-226.8)	107.3 (100.1-304.4)	0.481	114.1 (99.2-248.4)	109.0 (101.4-353.8)	0.230
IL-6 (pg/ml)^a^	43.5 (38.7-72.5)	43.8 (38.3-99.7)	0.313	42.1 (39.6-66.6)	43.4 (36.8-81.5)	0.584
MDA (μmol/l)^a^	6.0 (5.0-10.5)	6.3 (5.1-12.5)	0.296	6.0 (4.9-11.3)	6.5 (5.4-26.0)	0.289
CD −34^+^ (%)	0.72 ± 0.15	0.88 ± 0.20	0.101	0.77 ± 0.15	0.82 ± 0.21	0.488

TNF- α; Tumor necrosis factor alpha,IL-6; Interleukin-6, MDA; malondialdehyde

^a^Data was shown as Median (Inter Quartile Range) and analyzed by Non-Parametric Test and other were shown as mean ± Standard Deviation

There was a non-significant decrease in the fasting blood glucose and a significant decrease in HbA1c in *StemTech*^*TM*^ group at the end of the study compared to the baseline (Table 3). Safety of *StemTech*^*TM*^ was confirmed by other biochemical determinates.

**Table 3:** Comparison of biochemical assessments before and after of study among two studied groups

	Placebo		*P*-value	*StemTech* ^*TM*^		*P*-value
	Before	After		Before	After	
FBG (mg/dl)	179.9 ± 63.3	157.4 ± 39.9	0.217	187.9 ± 54.9	167.2 ± 66.2	0.079
HbA1c (%)	8.5 ± 1.8	8.2 ± 1.6	0.393	9.1 ± 1.8	8.3 ± 1.6	0.006
Triglyceride (mg/dl)	214.1 ± 106.9	155.7 ± 59.8	0.300	173.9 ± 74.8	176.5 ± 74.3	0.810
Total cholesterol (mg/dl)	174.9 ± 62.7	170.1 ± 42.9	0.657	174.9 ± 25.5	172.1 ± 27.0	0.646
LDL-C (mg/dl)	81.8 ± 40.7	94.9 ± 35.9	0.127	90.0 ± 20.9	89.5 ± 21.0	0.913
HDL-C (mg/dl)	40.5 ± 8.8	41.0 ± 9.6	0.780	43.6 ± 7.8	43.9 ± 9.2	0.840
CRP (mg/L)	2.7 ± 2.4	2.5 ± 3.0	0.621	2.9 ± 2.7	3.0 ± 2.5	0.347
AST (U/L)	22.1 ± 8.6	21.9 ± 8.3	0.927	23.4 ± 15.3	21.5 ± 9.7	0.334
ALT (U/L)	33.3 ± 26.1	29.3 ± 18.8	0.171	29.3 ± 13.7	26.7 ± 10.4	0.200
BUN (mg/dl)	25.9 ± 15.6	25.8 ± 15.2	0.950	22.8 ± 10.4	22.3 ± 8.3	0.807
Creatinine (mg/dl)	0.99 ± 0.19	0.95 ± 0.18	0.053	0.95 ± 0.16	0.93 ± 0.13	0.433

FBG; Fasting blood glucose, HbA1c; Glycated hemoglobin, LDL-C; Low-density lipoprotein Cholesterol, HDL-C; high- density lipoprotein Cholesterol, CRP; C- reactive protein, AST; aspartate aminotransferase, ALT; Alanine transaminase, BUN;blood urea nitrogen, WBC; white blood cell, RBC; red blood cell

Data are mean ± Standard Deviation and percent where indicated

Evaluation of hematologic factors in the placebo group showed a difference in white blood cells (WBCs). No further changes in other factors in both groups were noticed.

During the study there was only one person in the placebo group who complained about nausea and no other significant side effects were reported.

## Discussion

Diabetes not only reduces the value and extent of life, but also has enormous health-caring related costs. Global health expenditures for diabetes in 2030 will be 30–34 % more than 2010 [22]. The most recent WHO assessment shows that 171 million people worldwide are diabetic and this trend is going to be more than twice in the year 2030 [23]. There is a mounting concern to find a safe and effective treatment or nutrition component as a suitable supplement in order to cause the problem. *Spirulina,* a filamentous cyanobacteriumis an appropriate candidate, which United Nations World Food Conference confirmed Spirulina as “the best for tomorrow” [24].

Some beneficial effects of Blue-Green Alga extract on health of diabetic animal have been reported [25, 26]. Our main effort in this randomized clinical trial was to consider the role of *Stemtech*^*TM*^ on HbA1C, lipid profile, glycemic control, CD markers, as well as its side effects in patients with type 2 diabetes. We showed in patients with type-2 diabetes mellitus, *Stemtech*^*TM*^ diet could lower the HbA1c. Our results are on the same page with other studies which have measured the effect of blue-green algae on glucose levels in diabetic rats. The increased HbA1c is produced when the level of hemoglobin decreased and it leads to the highest level of blood glucose. Animals who were given Blue-Green Algae extract, which is a rich source of iron, had higher levels of hemoglobin, which is considered as the main cause of HbA1C level reduction [27]. They established that water-soluble fraction was useful in lowering the fasting serum glucose level [28]. And also they showed that rats, who lost their weight, after *Spirulina* treatment regained their body weight. A significant decrease in the fasting blood sugar level of patients after 21 days of 2 g/ day total *Spirulina* supplementation has been shown [16]. In another study, the oral administration of 15 mg/kg Spirulinafor 45 days in diabetic rats significantly reduced the blood glucose level [25]. There was an emphasize on crude extract and insulin-like protein of Spirulina instead of an aqueous and ethanolic extracts [29]. Liver levels of triglycerides and phospholipids positively and significantly responded in rats which was fed with a diet supplemented with 5 % Spirulina and either 60 % glucose or 60 % fructose [30].

Decreasing the blood glucose concentration in diabetic rats probably is done through stimulation of the β-cells of Langerhans islets to increase the production of insulin. The possible mechanism by which Spirulina produces its antihyperglycemic effects may be through potentiating of the pancreatic secretion of insulin from islet β-cell or due to enhancement of transport of blood glucose to the peripheral tissue [29]. This could possibly be due to the high fiber content of Blue-Green Alga that interferes with the glucose absorption [31] or probable action of producing polypeptides after digestion of Blue-Green Algae [16]. Several studies support that the mobilization, migration and differentiation of bone marrow stem cells in the target tissue constitute a natural phenomenon of healing in the human body [32–35].

Mean serum chemical parameters (Triglyceride, Total Cholesterol, LDL, HDL, CRP, AST, ALT, BUN and Creatinine) in treated and control groups before and after the study showed no significant differences which indicates the safety of Stemtech^TM^. These findings strengthened the results of other studies showing that even higher exposure to *StemEnhance* does not lead to toxicity [19, 36–38]. Levent Dirikolu in 2010 demonstrated that feeding StemEnhance at 600 mg/kg for two weeks was not toxic to Wistar rats. It also did not affect the behavior or physical assessments of the rats. The dose of *StemEnhance* tested in the rat was approximately equal to20 times higher than the maximum label-recommended daily dose for human use. Consequently, it would appear that no toxicological risk is associated to the use of *StemEnhance* at label doses [39]. Not only higher doses of Spirulina (up to 73 %) did not show any considerable difference in experimental and control animals, but it also produced an increase in the relative weight of some viscera [40]. Apparently AFA is well tolerated and good supply of polyunsaturated fatty acids [41, 42] which may increase the nutritional value of the diet.

Some studies have shown that the consumption of one gram of Stemtech^TM^ led to a considerable increase in the percentage of circulating CD34+ cells after one hour [19]. Also, they proved that mobilization of bone marrow CD34+ cells was related to L- selectin ligand contained in Stem Enhance which caused down regulation of CXCR4 chemokine receptor. The interruption of the binding of SDF-1 to CXCR4 chemokine receptor which leads to mobilization of CD34+ stem cells in rats is detectable between 2–4 weeks [43]. As we shown in Table 2 there was no significant differences in cell markers and antioxidant before and after intervention in both groups A and B and it goes without saying that our intervention period of 12 weeks was enough to see a difference.

Triglyceride, total cholesterol, LDL and HDL did not change significantly in our two groups before and after intervention, however, some studies showed that Blue-Green Algae inhibits intestinal cholesterol absorption and decreases the hepatic lipids and leads to attenuation of plasma total cholesterol, and triglyceride concentrations. The anti-inflammatory function of the BGA is mediated, at least in part, by inhibiting the Nuclear Factor Kappa B pathway to decrease the production of pro-inflammatory mediators. BGA can also decrease oxidative stress due to their free radical scavenging activity and inhibition of lipid per-oxidation [44]. According to the fact that cell marker, immunological parameters showed no significant changes statistically (Table 2), the power of each parameter is calculated, which was 60-70 % in Placebo and 15-20 % in *Stemtech*^*TM*^ groups, respectively. Therefore, it seems that the evidences for the effectiveness of *Stemtech*^*TM*^ on these parameters cannot be declined easily. In order to find more authentic results, we need some studies with larger sample size.

## Conclusion

Even though, hypoglycemic property of Blue-Green Algae extracts has been reported earlier, this study is the first randomized clinical trial of *Stemtech*^*TM*^ in a group of Iranian diabetic patients. *Stemtech*^*TM*^ intervention brought in favorable effects on HbA1c in our diabetic patients consequently suggest *Stemtech*^*TM*^ as a functional food in management of diabetes.
